# Stereoregular Brush Polymers and Graft Copolymers by Chiral Zirconocene-Mediated Coordination Polymerization of P3HT Macromers

**DOI:** 10.3390/polym9040139

**Published:** 2017-04-13

**Authors:** Yang Wang, Travis S. Bailey, Miao Hong, Eugene Y.-X. Chen

**Affiliations:** 1 Department of Chemistry, Colorado State University, Fort Collins, CO 80523-1872, USA; Yang.Wang@colostate.edu; 2 School of Fundamental Sciences, China Medical University, Shenyang 110122, China; 3 Department of Chemical and Biological Engineering, Colorado State University, Fort Collins, CO 80523-1370, USA; Travis.Bailey@colostate.edu; 4 State Key Laboratory of Organometallic Chemistry, Shanghai Institute of Organic Chemistry, Chinese Academy of Sciences, Shanghai 200032, China

**Keywords:** poly(3-hexylthiophene), zirconocene, stereoregular polymer, brush polymer, graft polymer

## Abstract

Two poly(3-hexylthiophene) (P3HT) macromers containing a donor polymer with a polymerizable methacrylate (MA) end group, P3HT-CH_2_-MA and P3HT-(CH_2_)_2_-MA, have been synthesized, and P3HT-(CH_2_)_2_-MA has been successfully homopolymerized and copolymerized with methyl methacrylate (MMA) into stereoregular brush polymers and graft copolymers, respectively, using chiral *ansa*-zirconocene catalysts. Macromer P3HT-CH_2_-MA is too sterically hindered to polymerize by the current Zr catalysts, but macromer P3HT-(CH_2_)_2_-MA is readily polymerizable via either homopolymerization or copolymerization with MMA in a stereospecific fashion with both *C*_2_-ligated zirconocenium catalyst **1** and *C*_s_-ligated zirconocenium catalyst **2**. Thus, highly isotactic (with *mm*% ≥ 92%) and syndiotactic (with *rr*% ≥ 93%) brush polymers, *it*-PMA-*g*-P3HT and *st*-PMA-*g*-P3HT, as well as well-defined stereoregular graft copolymers with different grafted P3HT densities, *it*-P(M)MA-*g*-P3HT and *st*-P(M)MA-*g*-P3HT, have been synthesized using this controlled coordination-addition polymerization system under ambient conditions. These stereoregular brush polymers and graft copolymers exhibit both thermal (glass and melting) transitions with *T*_g_ and *T*_m_ values corresponding to transitions within the stereoregular P(M)MA and crystalline P3HT domains. Acceptor molecules such as C_60_ can be effectively encapsulated inside the helical cavity of *st*-P(M)MA-*g*-P3HT to form a unique supramolecular helical crystalline complex, thus offering a novel strategy to control the donor/acceptor solar cell domain morphology.

## 1. Introduction

Poly(3-hexylthiophene) (P3HT) is an important conjugated donor polymer in the field of polymer electronic devices, such as organic field-effect transistors [1,2,3,4,5,6,7] and organic/polymeric photovoltaic (OPV) solar cells [8,9,10,11,12,13], thanks to its well-balanced all-around properties in terms of solution solubility, chemical stability, and charge mobility. These OPV devices are typically based on a bulk heterojunction fabricated from a blend of a donor (typically a conjugated polymer such as P3HT) and an acceptor (typically a fullerene such as C_60_ and its derivatives). Intense research and development on both new materials and new device architectures has resulted in gradually improved power conversion efficiencies of the OPV devices over the years, but it has been a challenge to precisely control the domain morphology of the donor/acceptor active layer (including size, geometry and phase segregation), which is one of the critically important parameters of the donor/acceptor assemblies determining the overall OPV device efficiency [8,9,14,15,16,17,18]. Owing to the solution-based deposition of the active layer, where two phases self-assemble from a physical blend into the solid film, one only has implicit control over the resulting morphology, typically *kinetically* controlled.

Furthermore, to fulfill the diverse requirements in organic electronic devices, the design of only a linear donor polymer structure has showed its limitations. In this context, copolymers of various architectures, such as block, graft, and branched polymers, are of interest due to their architectures that combine two or more dissimilar polymer components into one polymer with typically self-assembled nanostructures for tuning their properties for diverse application requirements [19,20,21,22]. Among such polymers, densely grafted polymers (or brush polymers) have far higher polymer chain mobilities, relative to linear polymers with comparable molecular weights due to the absence of chain entanglements [23,24]. Therefore, brush polymers of P3HT, which can combine the benefits of the brush architecture and the good electronic properties of the linear P3HT, are interesting materials for electronic devices. There are a few existing methods to synthesize P3HT-containing brush polymers, such as “grafting to” methods to graft P3HT to silicon surface [25,26] and “click reactions” to link P3HT to a polymer main chain [27,28]. However, these methods were met with some challenges, including the difficulty for the P3HT grafted to the silicon surface to self-assemble into the desired morphology and the need for the multistep synthesis involved in the click reaction route. 

We hypothesized that the metal-mediated controlled/living and stereospecific coordination-addition polymerization of polar vinyl monomers [29] such as methacrylates could provide not only a convenient synthesis of well-defined P3HT graft polymers under ambient conditions, but also unique stereoregular graft polymers, considering its high degree of control over polymerization characteristics, including stereochemistry. It is noted here that syndiotactic poly(methyl methacrylate) (*st*-PMMA) brush grafted on solid has been synthesized using a surface-initiated living anionic polymerization method [30]. Guided by this hypothesis, we designed two P3HT macromers with the polymerizable methacrylate end group, P3HT-CH_2_-MA and P3HT-(CH_2_)_2_-MA, subject to the metal-mediated coordination polymerization. Two neutral zirconocene complexes (pre-catalysts), *C*_2_-ligated *ansa*-zirconocene bis(ester enolate) *rac-*C_2_H_4_(*η*^5^-indenyl)_2_Zr[OC(O*^i^*Pr)=CMe_2_]_2_ (Zr-**1**) [31] and *C*_s_-ligated *ansa*-zirconocene bis(ester enolate) [Ph_2_C(Cp)(Flu)]Zr[OC(O*^i^*Pr)=CMe_2_]_2_ (Zr-**2**; Cp = *η*^5^-cyclopentadienyl, Flu = *η*^n^-fluorenyl) [32,33], which can be readily activated with [Ph_3_C][B(C_6_F_5_)_4_] via hydride abstraction, followed by Michael addition, to generate the corresponding cationic complexes (catalysts) Zr-**1^+^**[B(C_6_F_5_)_4_]^−^ and Zr-**2^+^**[B(C_6_F_5_)_4_]^−^, respectively [31,32,33], were chosen to investigate the polymerizability and polymerization behavior of the two macromers. Accordingly, this contribution describes the synthesis of the two P3HT macromers and successful polymerization of P3HT-(CH_2_)_2_-MA into stereoregular brush polymers and graft copolymers, therefore achieving the first successful synthesis of brush-like polymers with the stereoregular P(M)MA as the main chain and P3HT grafting as side chains, by the metal-mediated coordination polymerization. Moreover, utilizing the unique ability of *st*-PMMA to encapsulate fullerenes by forming helical crystalline inclusion complexes [34,35], we have shown that the graft copolymer *st*-P(M)MA-*g*-P3HT can also effectively encapsulate C_60_ molecules in its helical cavity to form a supramolecular helical crystalline complex, thereby offering a unique example of a self-assembled donor/acceptor brush polymer active-layer domain morphology through the thermodynamically controlled, molecular recognition mechanism.

## 2. Materials and Methods

### 2.1. General Information

All synthesis and manipulations with air- and moisture-sensitive chemicals and reagents were performed using standard Schlenk techniques on a dual-manifold Schlenk line or in an inert gas (Ar or N_2_)-filled glovebox. NMR spectra were recorded on a Varian Inova 400 MHz or 500 MHz spectrometer (Varian, Palo Alto, CA, USA). Benzene-*d*_6_ and toluene-*d*_8_ were dried over sodium/potassium alloy and vacuum-distilled or filtered. Chemical shifts were referenced to residual undeuterated solvent resonances and are reported as parts per million relative to SiMe_4_. HPLC-grade organic solvents were first saturated with nitrogen during filling of the 20 L solvent reservoirs and then dried by passage through activated alumina (for Et_2_O, THF, and CH_2_Cl_2_) followed by passage through Q-5 supported copper catalyst (for toluene and hexanes) stainless steel columns.

Polymer weight-average molecular weights (*M*_w_) and molecular weight distributions or dispersities (*Đ* = *M*_w_/*M*_n_) were measured by gel permeation chromatography (GPC) analyses carried out at 40 °C and a flow rate of 1.0 mL/min, with DMF as the eluent on a Waters University 1500 GPC instrument (Waters, Milford, MA, USA), equipped with one PLgel 5 μm guard and three PLgel 5 μm mixed-C columns (Polymer Laboratories; linear range of M_W_ = 200–2,000,000). The instrument was calibrated with 10 PMMA standards, and chromatograms were processed with Waters Empower software (version 2002, Waters, Milford, MA, USA). Glass transition temperatures (*T*_g_) and melting-transition temperatures (*T*_m_) of the polymers were measured by differential scanning calorimetry (DSC) on a Q20 DSC, TA Instruments, New Castle, DE, USA. Samples were first heated until 250 °C at 10 °C·min^−1^, cooled to 25 °C at 10 °C·min^−1^, and then reheated again at 10 °C·min^−1^ to 250 °C. All *T*_g_ and *T*_m_ values were obtained from the second heating scan, after removing the thermal history. The tacticity of the polymers was analyzed by ^1^H and ^13^C-NMR based on that of PMMA. The macromer samples were analyzed by matrix-assisted laser desorption/ionization time-of-flight mass spectroscopy (MALDI-TOF MS); the experiment was performed on a Ultraflex MALDI-TOF mass spectrometer (Bruker Daltonics, Billerica, MA, USA) operated in positive ion, reflector mode using a Nd: YAG laser at 355 nm and 25 kV accelerating voltage. External calibration was done using a peptide calibration mixture (4–6 peptides) on a spot adjacent to the sample. The raw data were processed in the FlexAnalysis software (version 2.4, Bruker Daltonics).

Reagents 2,5-dibromo-3-hexylthiophene, *t*-BuMgCl, 9-borabicyclo[3.3.1]nonane (9-BBN), 1,2-bis(diphenylphosphino)ethane nickel(II) chloride [Ni(dppp)Cl_2_], tris(dibenzylideneacetone)dipalladium [Pd_2_(dba)_2_], tri-*tert*-butylphosphine [P(t-Bu)_3_], and tri-*n*-butyl(vinyl) tin were purchased from Sigma-Aldrich (St. Louis, MO, USA) and used as received. Methacryloyl chloride was purchased from Sigma-Aldrich, distilled and stored at −40 °C in the glovebox for further use. Phosphorus oxychloride (POCl_3_), sodium borohydride, triethylamine, hydrogen peroxide and sodium hydroxide were purchased from TCI America and used as received. C_60_ was purchased from Sigma-Aldrich and dried under vacuum at 60 °C for further use. Methyl methacrylate (MMA) was purchased from Alfa Aesar Chemical Co. (Ward Hill, MA, USA), dried over activated CaH_2_ overnight, followed by vacuum distillation; it was further purified by titration with neat tri(*n*-octyl)aluminum to a yellow end point, followed by distillation under reduced pressure. The purified monomer was stored in a brown bottle at −30 °C inside of a glovebox freezer. Zirconocene pre-catalysts Zr-**1** and Zr-**2** as well as their activated cationic catalysts Zr-**1^+^**[B(C_6_F_5_)_4_]^−^ and Zr-**2^+^**[B(C_6_F_5_)_4_]^−^ were prepared according to literature procedures [31,32,33].

### 2.2. Synthesis of P3HT with H/Br Chain-Ends, H-P3HT-Br

Literature procedures [36] were modified to prepare the H/Br end-capped P3HT. 2,5-Dibromo-3-hexylthiophene (1.5 g, 4.6 mmol), *t*-BuMgCl (4.41 mmol) and THF (9 mL) were added to a 100 mL Schlenk flask and the mixture was stirred at room temperature for 20 h. The obtained yellow solution was transferred to 100 mL Schlenk flask which contained Ni(dppp)Cl_2_ (40.5 mg in 20 mL THF) using cannula and the resulting mixture was stirred for 12 min. The polymerization was stopped by addition of 5 mL of 5.0 M HCl. After precipitation in methanol, the polymer was filtered into a Soxhlet thimble and extracted with methanol and hexanes overnight to wash away the residual monomer and low molecular weight impurities. The purified polymer was obtained in 70% yield by redissolution in CHCl_3_ and precipitation in methanol.

**Figure 1:**
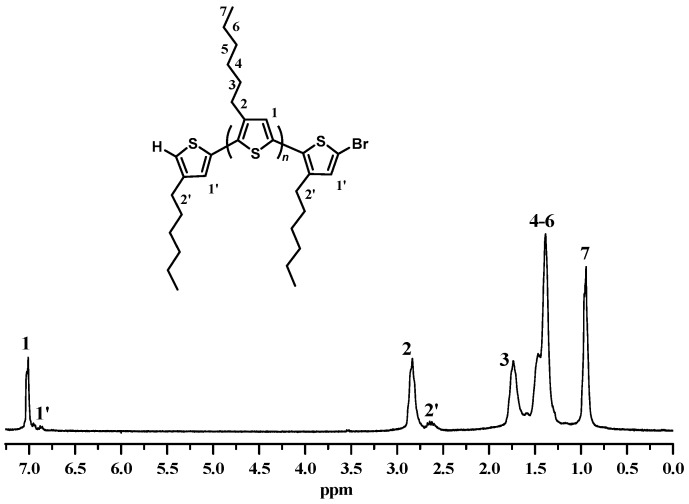
^1^H-NMR (400 MHz, CDCl_3_) spectrum of H-P3HT-Br with *M*_w_ ~ 3500 g/mol.

^1^H-NMR spectrum of P3HT with H/Br chain-ends (H-P3HT-Br) is shown in Figure 1. Except the characteristic peaks of the P3HT main chain, the chain ends can also be observed at 6.7 and 2.8 ppm, attributed to the proton of the chain end’s double bond and the protons adjacent to the thiophene ring. The number-average molecular weight (*M*_n_) can be calculated according to the following formula: *M*_n_(NMR) = (2I_2.80ppm_/I_2.60ppm_) × 166 + 167 + 246. Accordingly, the *M*_W_ of H-P3HT-Br was calculated to be approximately 5000 and 3500 g/mol for the samples prepared by using the monomer/Ni ratio of 62/1 and 26/1, respectively.

### 2.3. Synthesis of Macromer P3HT-(CH_2_)_2_-MA 

H-P3HT-Br (0.52 g) was thoroughly dried overnight and transferred to a 250 mL Schlenk flask in a glovebox. Then Pd(dba)_3_ (20 mg), tri(*t*-butyl)phosphine (180 mg), tri-*n*-butyl(vinyl) tin (0.475 g) and THF (20 mL) were introduced. The flask was sealed, taken out of the glovebox, interfaced to a Schlenk line, and stirred at 55 °C overnight. After that, the reaction mixture was poured into methanol, filtered, and washed with methanol several times. The obtained crude product was dissolved in CHCl_3_, filtered with Kieselguhr to remove the Pd catalyst and then precipitated in methanol. The resulted P3HT-vinyl polymer was dried under vacuum at room temperature to a constant weight (yield: 95%). ^1^H-NMR (400 MHz; CDCl_3_), δ: 6.96 (s), 5.52 (d, –C*H*=CH_2_ chain-end), 5.10 (d, –CH=C*H*_2_ chain-end), 2.73 (m), 1.62 (m), 1.36 (m), 1.27 (m), 0.84 (t) ppm.

P3HT-vinyl (0.25 g) and 15 mL THF were added to a 100 mL dried Schlenk flask and the mixture was heated to 60 °C with stirring until P3HT-vinyl was dissolved. 9-BBN (0.5 M in THF, 3.5 mL) was added via syringe and the solution was allowed to stir at 60 °C until the double bond signals (via ^1^H-NMR monitoring) completely disappeared. This process usually needed 30 h to reach a full conversion. After that, 6 M (1 mL) NaOH was added and the mixture was stirred for 15 min. After cooling to room temperature, 3 mL H_2_O_2_ (30%) was added, and the mixture was heated to 45 °C and reacted for another 24 h. Following precipitation in water-methanol mixture, filtration, washing and dried, the pure P3HT-(CH_2_)_2_-OH was obtained in 92.7% yield. ^1^H-NMR (400 MHz; CDCl_3_), δ: 6.96 (s), 3.89 (s, –C*H*_2_OH chain-end), 2.73 (m), 1.62 (m), 1.36 (m), 1.27 (m), 0.84 (t) ppm.

P3HT-(CH_2_)_2_-OH (0.25 g) and 15 mL THF were added to a 100 mL dried Schlenk flask and the mixture was heated to 40 °C until P3HT-(CH_2_)_2_-OH was dissolved. Triethylamine (3 mL) and methacryloyl chloride (2 mL) were then introduced and stirred at 40 °C for 24 h prior to precipitation in methanol. The resulting final product P3HT-(CH_2_)_2_-MA was filtered, washed and dried in vacuo at room temperature to a constant weight (yield: 97.1%). ^1^H-NMR (400 MHz; CDCl_3_), δ: 6.98 (s), 6.17 (s, =CH_2_ chain end), 5.60 (s, =CH_2_ chain end), 4.34 (m, CH_2_ chain end), 3.13 (m, CH_2_ chain end), 2.81 (t), 1.98 (s, Me chain end), 1.70 (m), 1.44 (m), 1.35 (m), 0.92 (t) ppm.

### 2.4. Synthesis of P3HT-CH_2_-MA

H-P3HT-Br (1.0 g) was dried overnight and dissolved in 260 mL of toluene under nitrogen. DMF (5.12 mL, 66.3 mmol) and phosphorus (V) oxychloride (POCl_3_) (5.30 mL, 58 mmol) were then added to the solution. The reaction mixture was stirred at 75 °C for 24 h, after which the solution was cooled down to room temperature, followed by the addition of a saturated aqueous solution of sodium acetate. The mixture was stirred for 4 h, and then the polymer product was extracted with CHCl_3_, followed by precipitation into cold methanol and washed with cold *n*-hexane. After drying under vacuum, P3HT-CHO was obtained in 93% yield. ^1^H-NMR (400 MHz, CDCl_3_), δ: 10.03 (s, CHO chain-end), 6.98 (s), 2.80 (t), 1.69 (m), 1.44 (m), 1.35 (m), 0.93 (t) ppm.

P3HT-CHO (0.5 g) was dissolved in 30 mL of THF under nitrogen, and NaBH_4_ (41.8 mg) was then added. The mixture was kept stirring at room temperature for 2 h, after which time the solvent was evaporated under vacuum. The resulting polymer was precipitated in cold methanol. After drying under vacuum, P3HT-CH_2_-OH was obtained in 96% yield. ^1^H-NMR (400 MHz, CDCl_3_), δ: 6.98 (s), 4.79 (d, C*H*_2_OH chain-end), 2.80 (t), 1.69 (m), 1.43 (m), 1.35 (m), 0.91 (t) ppm.

P3HT-CH_2_-OH (0.5 g) and 30 mL THF were added to a 100 mL dried Schlenk flask and the mixture was heated to 40 °C until P3HT-CH_2_-OH was dissolved. Triethylamine (6 mL) and methacryloyl chloride (4 mL) were then introduced and the resulting mixture was stirred at 40 °C for 24 h prior to precipitation in methanol. The resulting product P3HT-CH_2_-MA was filtered, washed and dried in vacuo at room temperature to a constant weight (yield: 95.3%). ^1^H-NMR (400 MHz; CDCl_3_), δ: 6.98 (s), 6.15 (s, =CH_2_ chain end), 5.60 (s, =CH_2_ chain end), 5.29 (s, –OCH_2_ chain end), 2.80 (t), 1.97 (s, Me chain end), 1.70 (m), 1.42 (m), 1.35 (m), 0.91 (t) ppm.

### 2.5. General Polymerization Procedures

Polymerizations were performed in 20 mL oven-dried glass reactors inside a glovebox. For homopolymerizations, a Zr pre-catalyst was premixed with an equimolar amount of activator [Ph_3_C][B(C_6_F_5_)_4_] in toluene for ca. 10 min to generate the corresponding activated cationic species. The polymerization was started by rapid addition of a macromer (P3HT-(CH_2_)_2_-MA or P3HT-CH_2_-MA) solution in toluene. After the prescribed time, the polymerization was immediately quenched by pouring the reaction solution into 5% HCl-acidified methanol. The polymer was collected by filtering, washing several times with methanol, and drying in a vacuum oven at 50 °C to a constant weight. For copolymerizations, a Zr pre-catalyst was premixed with an equimolar amount of activator [Ph_3_C][B(C_6_F_5_)_4_]. The polymerization was started by rapidly adding the solution of macromer and comonomer MMA in toluene. After a prescribed time, the polymerization was immediately quenched by pouring the reaction solution into 5% HCl-acidified methanol. The crude polymer precipitated from methanol was dissolved in DMF to separate the P(M)MA-*g*-P3HT copolymer (soluble) and unpolymerized P3HT-MA (insoluble). The DMF-soluble polymer was then precipitated in methanol, filtered, washed several times with methanol, and dried in a vacuum oven at 50 °C to a constant weight.

### 2.6. Encapsulation of Acceptor C_60_ by st-P(M)MA-g-P3HT

Syndiotactic graft copolymer *st*-P(M)MA-*g*-P3HT (10 mg) was dissolved in a toluene solution of C_60_ (2 mg/mL, 1 mL) at 110 °C. After the solution was slowly cooled to room temperature (ca. 20 °C) without disturbance, the solution gradually gelled. The obtained soft gel was centrifuged for 10 min and the supernatant containing unencapsulated C_60_ was removed from the gel by careful decantation. The condensed gel was then washed with toluene and the solvent was removed by decantation after centrifugation. This procedure was repeated several times until no purple color of C_60_ retained. The solvent of complex gel was evaporated under vacuum at 50 °C to a constant weight.

## 3. Results and Discussion

### 3.1. Synthesis of Macromers P3HT-(CH_2_)_2_-MA and P3HT-CH_2_-MA

**Scheme 1:**
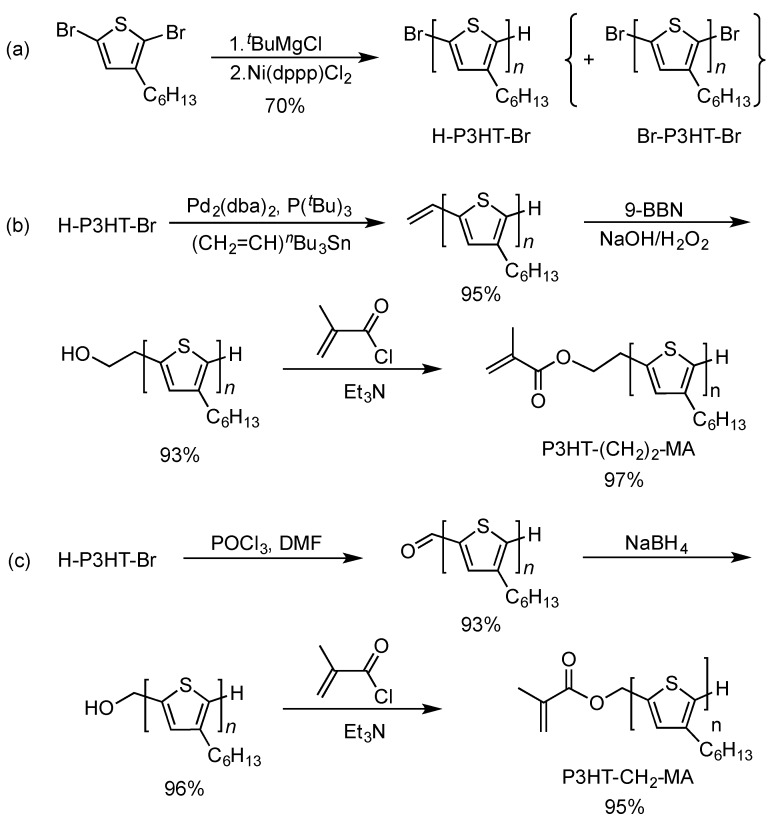
Synthetic routes to macromers P3HT-(CH_2_)_2_-MA and P3HT-CH_2_-MA.

**Figure 2:**
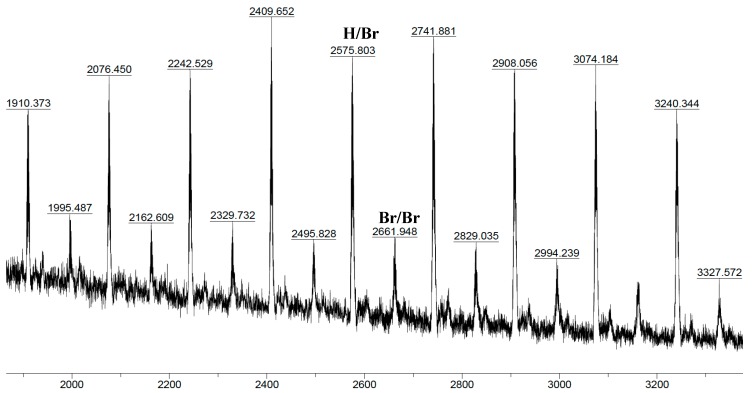
MALDI-TOF MS spectrum showing P3HT with both H/Br (major) and Br/Br (minor) chain-ends.

Two polymerizable P3HT-methacrylate macromers, P3HT-(CH_2_)_2_-MA and P3HT-CH_2_-MA, were synthesized following the routes outlined in Scheme 1b,c, starting from P3HT with H/Br chain-ends, H-P3HT-Br. This starting oligomeric material was prepared using the known quasi-living Grignard metathesis polymerization of 2,5-dibromo-3-hexylthiophene, which was first treated with *^t^*BuMgCl and followed by addition of initiator Ni(dppp)Cl_2_ (Scheme 1a) [36]. The formation of H-P3HT-Br was verified by ^1^H-NMR (Figure 1), from which the molecular weight (MW) was calculated to be approximately 5000 and 3500 g/mol for the samples prepared by using the monomer/Ni ratio of 62/1 and 26/1, respectively. MALDI-TOF MS (Figure 2) also showed the presence of a small amount of P3HT with Br/Br chain-ends, but no H/H “dead” chain-ends.

**Figure 3:**
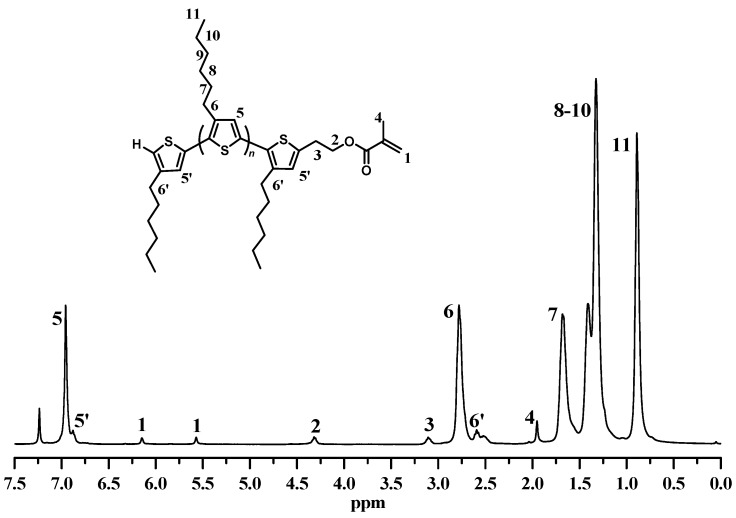
^1^H-NMR (400 MHz, CDCl_3_) spectrum of macromer P3HT-(CH_2_)_2_-MA.

**Figure 4:**
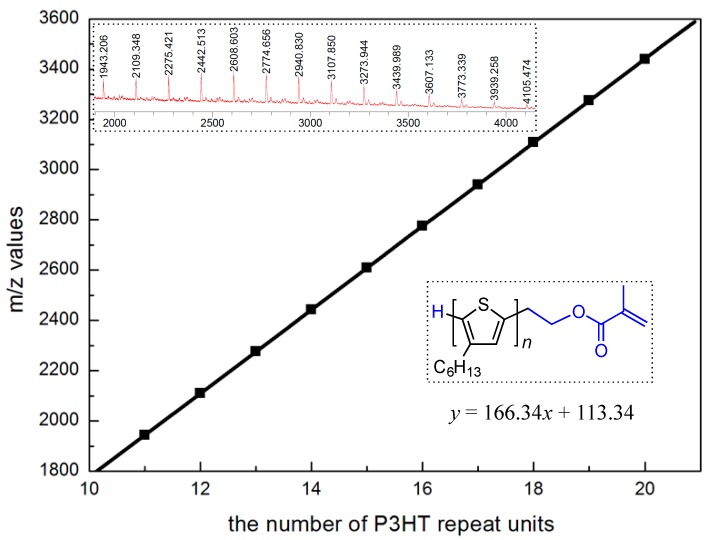
MALDI-TOF spectrum (inset) of macromer P3HT-(CH_2_)_2_-MA (*M*_w_ ~ 3500 g/mol by NMR) and plot of *m*/*z* values (*y*-axis) vs. the number of P3HT repeat units (*x*-axis).

Next, two different types of effective end-functionalization reactions were carried out utilizing the active bromide end group on the thiophene ring. First, the Stille coupling reaction of H-P3HT-Br with *^n^*Bu_3_Sn(CH=CH_2_) in the presence of Pd_2_(dba)_2_ as the catalyst led to the vinyl end-functionalized H-P3HT-(CH=CH_2_) in 95% isolated yield, which was subsequently converted into hydroxyl end-functionalized H-P3HT-(CH_2_CH_2_OH) in 93% isolated yield via hydroboration-oxidation reaction with 9-BBN/NaOH/H_2_O_2_. In the final step, the acylation of this hydroxyl end-functionalized P3HT intermediate with methacryloyl chloride in the presence of Et_3_N afforded macromer P3HT-(CH_2_)_2_-MA in 97% isolated yield (Scheme 1b). It is worth noting that a small amount of the P3HT with doubly functionalized chain ends also formed during the process (due to the initial presence of Br-P3HT-Br) was readily removed after a simple purification procedure involving washing with methanol. The entire course of the reaction and the formation of the isolated intermediates were monitored and confirmed by ^1^H-NMR. For example, Figure 3 depicts the spectrum of the final macromer P3HT-(CH_2_)_2_-MA, clearly showing the resonances for the two vinylidene protons >C=CH_2_ at δ 6.17 and 5.60 ppm (labeled as protons 1) and for the –OCH_2_CH_2_– group at δ 4.34 and 3.13 ppm (labeled as protons 2 and 3, respectively). The calculated *M*_n_ by ^1^H-NMR was ~3500 g/mol. The formation of the exclusive H/MA chain-end groups and the *M*_n_ were further confirmed by MALDI-TOF MS (Figure 4), which displays only one series of molecular mass ion peaks to show the presence of only one type of chain ends. A plot of *m*/*z* values vs. the number of P3HT repeat units yields a straight line with a slope of 166.3 and an intercept of 113.3 (Figure 4). Thus, the slope corresponds to the mass of the P3HT monomer, whereas the intercept is the sum of the masses for the chain-end groups, H/MA. Through the same synthetic procedure, P3HT-(CH_2_)_2_-MA with *M*_n_ ~5000 g/mol) was also synthesized.

The second type of the end-functionalization reaction began with the Vilsmeier-Haack formylation [37], followed by reduction and acylation (Scheme 1c). Thus, formylation of H-P3HT-Br with POCl_3_ led to H-P3HT-CHO in 93% isolated yield; subsequent reduction with NaBH_4_ afforded the corresponding H-P3HT-CH_2_OH in 96% isolated yield, which was acylated to the final macromer P3HT-CH_2_-MA in 95% isolated yield, after purification by removing any doubly functionalized P3HT possibly formed. ^1^H-NMR resonances characteristic of the methacryl end group, H_2_C=C(Me)C(=O)OCH_2_–, were observed at δ 6.15 and 5.60 ppm (s, H_2_C=), 5.29 (s, CH_2_), and 1.97 (s, Me), confirming the exclusive H/MA chain ends in the macromer.

### 3.2. Homopolymerization of P3HT-MA Macromers and Theirs Copolymerization with MMA

**Scheme 2:**
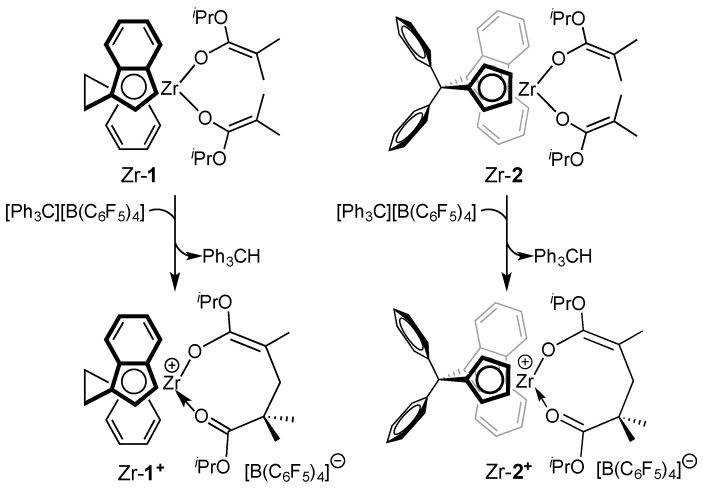
Structures of *C*_2_-symmetric and *C*_s_-symmetric pre-catalysts and activation reactions to the cationic catalysts.

To examine the polymerizability of the two P3HT-MA macromers, *C*_2_-ligated precatalysts Zr-**1** and *C*_s_-ligated Zr-**2**, which upon activation with [Ph_3_C][B(C_6_F_5_)_4_] generates the corresponding cationic catalysts Zr-**1^+^**[B(C_6_F_5_)_4_]^−^ and Zr-**2^+^**[B(C_6_F_5_)_4_]^−^ (Scheme 2) [31,32,33], were employed for the polymerization study. The results of their homopolymerization and copolymerization with MMA are summarized in Table 1. With *C*_2_-ligated catalyst Zr-**1^+^**[B(C_6_F_5_)_4_]^−^, macromer P3HT-(CH_2_)_2_-MA was successfully homopolymerized to the corresponding *isotactic* polymer in 92% yield (run 1). The resulting polymer has an isotactic poly(methacrylate) backbone and a P3HT side chain on every methacrylate repeat unit, thus a densely grafted polymer, or a brush polymer, *it*-PMA-*g*-P3HT, which displayed both glass transition temperature (*T*_g_) and melting-transition temperature (*T*_m_) endothermic peaks on the DSC curve characteristic of the isotactic poly(methacrylate) and crystalline P3HT domains, respectively (vide infra). Likewise, macromer P3HT-(CH_2_)_2_-MA was also successfully polymerized by *C*_s_-ligated catalyst Zr-**2^+^**[B(C_6_F_5_)_4_]^−^ to the corresponding syndiotactic brush polymer in 96% yield (run 4). The resulting densely grafted polymer *st*-PMA-*g*-P3HT exhibited also both the *T*_g_ and *T*_m_ values corresponding to the syndiotactic poly(methacrylate) and crystalline P3HT domains, respectively (vide infra). In sharp contrast, macromer P3HT-CH_2_-MA was not polymerizable by either *C*_2_- or *C*_s_-ligated catalysts, even after extended times (up to 12 h), attributable to the sterically hindered ester group of the macromer with only one carbon linkage between the P3HT chain and the MA moiety, which presumably hinders the coordination of this bulkier monomer to the cationic Zr center, the step essential for initiation of this coordination-addition polymerization.

**Figure 5:**
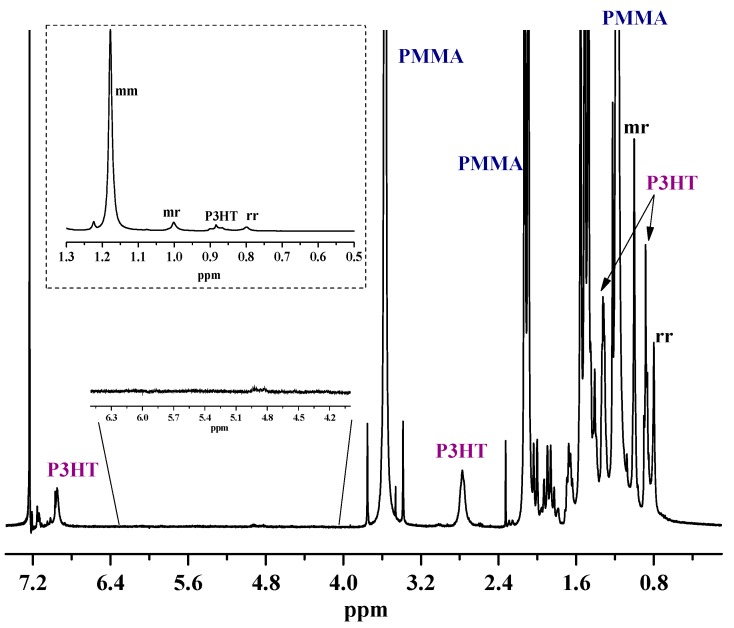
^1^H-NMR spectrum (400 MHz, in CDCl_3_) of graft copolymer *it*-P(M)MA-*g*-P3HT produced by *C*_2_-ligated Zr-**1** (Table 1, Run 3). Insets: blowup of the PMMA methyl triad region (top) and P3HT-(CH_2_)_2_-MA double bond region (7×, bottom).

**Figure 6:**
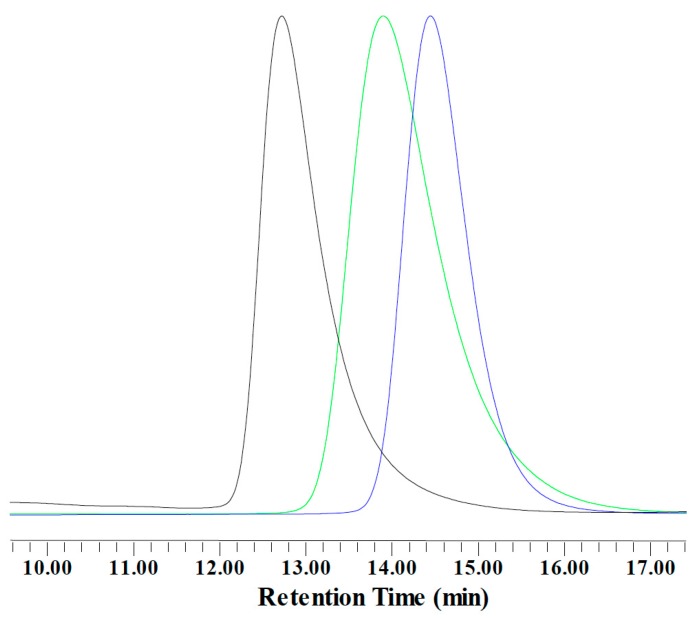
GPC curves for stereoregular graft copolymers *it*- or *st*-P(M)MA-*g*-P3HT: black line (Table 1, Run 2); blue line (Table 1, Run 3); and green line (Table 1, Run 5).

Owing to the overlap between the ^1^H-NMR resonances of the hexyl group of P3HT and those of the methyl triads (*mm*, *mr* and *rr* appeared at δ 1.20, 1.02 and 0.82 ppm) of the poly(methacrylate) of the homopolymers, we were unable to obtain the accurate tacticity values of these homopolymers, although it was estimated to be at least >90%. However, this issue was addressed through our study of copolymerizations with MMA. First, the copolymerization of P3HT-(CH_2_)_2_-MA with MMA was investigated by *C*_2_-ligated Zr-**1^+^**[B(C_6_F_5_)_4_]^−^ with two different MMA/macromer ratios; the relatively high 383/1 MMA/macromer ratio run afforded a high *M*_w_ isotactic graft copolymer *it*-P(M)MA-*g*-P3HT with *M*_w_ = 132 kg/mol, *Ð* = 1.25, and isotacticity *mm*% = 92% (run 2). Again, the thermal transitions are consistent with the isotactic poly(methacrylate) and crystalline P3HT domains (vide infra). As expected, the graft density is relatively low, with only 4.3 mol % P3HT incorporation. The graft density can be increased by decreasing the MMA/macromer ratio (or increasing the macromer in the feed); thus isotactic graft copolymer with 13.2 mol % P3HT was produced with a MMA/macromer ratio of 38/1 (run 3). This feed ratio change also enhanced the isotacticity of the graft copolymer slightly to 94%, while the *T*_g_ and *T*_m_ values were essentially the same (run 3 vs. 2). Figure 5 depicts the ^1^H-NMR spectrum of *it*-P(M)MA-*g*-P3HT, showing that the characteristic peaks of the PMMA main chain and P3HT side chain appeared simultaneously after the copolymerization, while the peaks at 5.60 and 6.17 ppm due to the double bond of the MA end disappeared (see the inset with 7× intensity enhancement). GPC traces of the above two graft copolymers showed unimodal molecular weight distributions with low *Ð* values from 1.14–1.25 (Figure 6). In short, these results demonstrated the copolymerization proceeded successfully to form the well-defined isotactic graft copolymer. 

Second, the copolymerization of P3HT-(CH_2_)_2_-MA with MMA was also examined by using the *C*_s_-ligated catalyst Zr-**2^+^**[B(C_6_F_5_)_4_]^−^ with three different MMA/macromer ratios, 383/1 (run 5), 101/1 (run 6), and 27/1 (run 7), enabling the synthesis of syndiotactic graft copolymer *st*-P(M)MA-*g*-P3HT with the graft density varying from 3.9, 8.0, and 14.5 mol % P3HT, respectively. Noteworthy is the high syndiotacticity for all the graft copolymers produced (*rr*% = 93–95%). The DSC (run 5, Table 1) and GPC (run 5, Table 1; Figure 6) results are also consistent with the formation of well-defined syndiotactic graft copolymer *st*-P(M)MA-*g*-P3HT.

### 3.3. Thermal Behavior of Brush Polymers and Graft Copolymers and Inclusion Complex Formation between C_60_ and st-P(M)MA-g-P3HT

**Figure 7:**
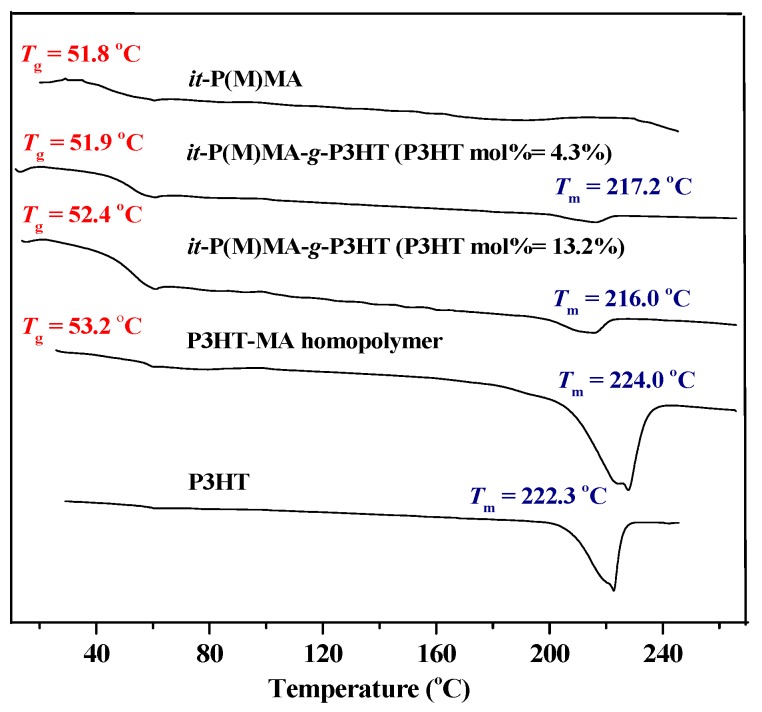
DSC curves for isotactic brush homopolymer *it*-PMA-*g*-P3HT and graft copolymer *it*-P(M)MA-*g*-P3HT (4.3 mol % and 13.2 mol % P3HT) produced by the *C*_2_-ligated catalyst. Curves for isotactic *it*-PMMA and P3HT are included for comparison.

**Figure 8:**
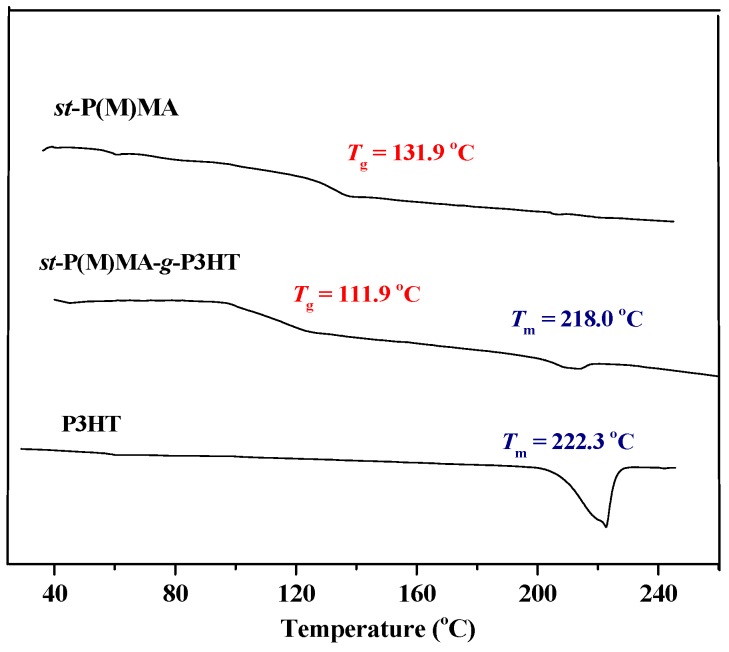
DSC curves for graft copolymer *st*-P(M)MA-*g*-P3HT (3.9 mol % P3HT, run 5, Table 1) produced by the *C*_s_-ligated catalyst. Curves for syndiotactic *st*-PMMA and P3HT are included for comparison.

The thermal transition temperatures of brush homopolymers and graft copolymers were measured by DSC analysis. Typical second heating scans of DSC curves are shown in Figure 7 and Figure 8, displaying both *T*_g_ and *T*_m_ for both isotactic and syndiotactic graft homo- and copolymers. As a control, homopolymers *it*-PMMA, *st*-PMMA, and P3HT prepared by the current catalyst systems were also analyzed by DSC to show their thermal transitions: *T*_g_ = 51.8 °C for *it*-PMMA, *T*_g_ = 132 °C for *st*-PMMA, and *T*_m_ = 222 °C for P3HT. It is clear from the DSC trace (Figure 7) that isotactic brush polymer *it*-PMA-*g*-P3HT obtained from the homopolymerization of macromer P3HT-(CH_2_)_2_-MA by the *C*_2_-ligated catalyst exhibited both thermal transitions with *T*_g_ = 53.2 °C and *T*_m_ = 224 °C, characteristic of the isotactic poly(methacrylate) and crystalline P3HT domains, respectively, which further demonstrated the successful homopolymerization. For graft copolymer *it*-P(M)MA-*g*-P3HT, both thermal transitions were also observed (Figure 7). Thus, *it*-P(M)MA-*g*-P3HT with the relatively low graft density of 4.3 mol % P3HT, the *T*_g_ and *T*_m_ values were 51.9 °C and 217 °C, corresponding to the isotactic PMMA and crystalline P3HT domains, respectively. Increasing the graft density to 13.2 mol % P3HT kept the *T*_g_ and *T*_m_ values essentially unchanged.

Likewise, syndiotactic brush polymer *st*-PMA-*g*-P3HT obtained from the homopolymerization of macromer P3HT-(CH_2_)_2_-MA by the *C*_s_-ligated catalyst also exhibited both thermal transitions with *T*_g_ = 133 °C and *T*_m_ = 219 °C, corresponding to the syndiotactic poly(methacrylate) and crystalline P3HT domains, respectively. For the syndiotactic graft copolymer *st*-P(M)MA-*g*-P3HT, the observed *T*_g_ of 112 °C and *T*_m_ of 218 °C (Figure 8) are attributed to the syndiotactic PMMA main chain and crystalline P3HT side chain domains, again indicating the successful copolymerization of P3HT-(CH_2_)_2_-MA and MMA.

**Figure 9:**
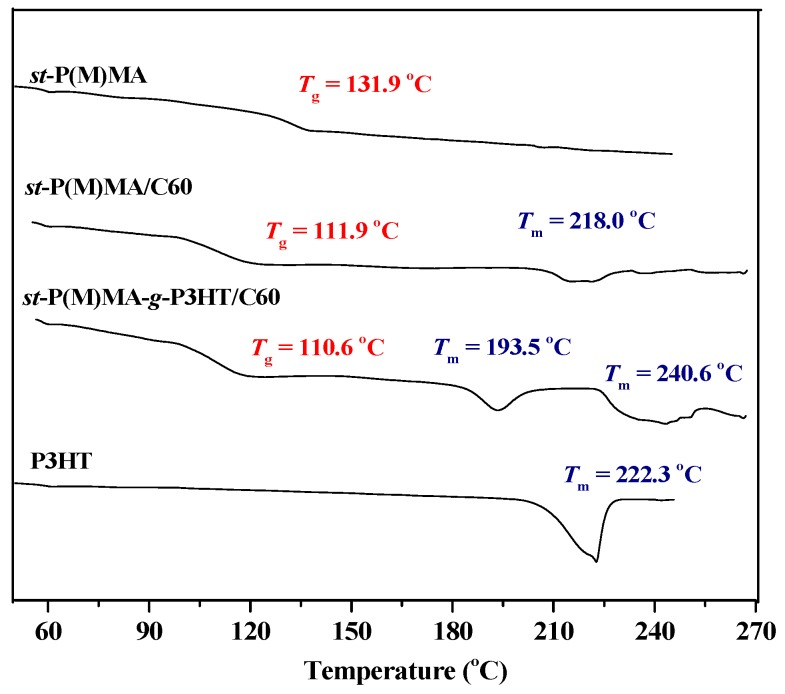
DSC curves for *st*-P(M)MA-*g*-P3HT/C_60_ complex. Curves for syndiotactic *st*-PMMA and P3HT are included for comparison.

Considering that *st*-PMMA possesses the unique ability to encapsulate fullerenes such as C_60_ within its large (~1 nm) helical cavity to form a peapod-like helical crystalline inclusion complex [24] we explored the possibility of syndiotactic graft copolymer *st*-P(M)MA-*g*-P3HT to form a crystalline inclusion complex with C_60_. Thus, the graft copolymer was dissolved in a toluene solution of C_60_ at 110 °C; after the solution was slowly cooled to room temperature without disturbance, the solution gradually gelled. The strong evidence for successful encapsulation of C_60_ with the helical hollow space of *st*-P(M)MA-*g*-P3HT was provided by DSC analysis (Figure 9). For *st*-PMMA-*g*-P3HT/C_60_ inclusion complex, in addition to the *T*_g_ of 114 °C for the *st*-PMMA main chain and *T*_m_ of 194 °C for the crystalline P3HT side chain, a new *T*_m_ peak appeared at 241 °C attributed to the crystalline inclusion complex formation. As a control, isotactic graft copolymer *it*-P(M)MA-*g*-P3HT did not encapsulate C_60_ to form an inclusion complex, consistent with the inability of *it*-PMMA to encapsulate C_60_ [34,35].

## 4. Conclusions

In summary, we have designed and synthesized two P3HT macromers with the polymerizable methacrylate end group. Although macromer P3HT-CH_2_-MA was found to be too sterically hindered to polymerize by the current chiral *ansa*-zirconocene-based catalysts via the coordination-addition polymerization mechanism, macromer P3HT-(CH_2_)_2_-MA with eased steric pressure through insertion of one more carbon to the linkage between the P3HT and MA moieties was readily homopolymerized or copolymerized with MMA in the stereospecific fashion. With the *C*_2_-ligated zirconocenium catalyst **1**, highly isotactic brush polymer *it*-PMA-*g*-P3HT and graft copolymer *it*-P(M)MA-*g*-P3HT with *mm*% ≥ 92% were readily synthesized under ambient conditions. The P3HT graft density in the graft copolymer can be controlled by the initial MMA/macromer feed ratio. The synthesized isotactic brush polymer and graft copolymers displayed both thermal transitions with the *T*_g_ and *T*_m_ values corresponding to the isotactic P(M)MA and crystalline P3HT domains. On the other hand, the *C*_s_-ligated zirconocenium catalyst **2** produced highly syndiotactic brush polymer *st*-PMA-*g*-P3HT and graft copolymer *st*-P(M)MA-*g*-P3HT with *rr*% ≥ 93%. The resulting syndiotactic brush polymer and graft copolymers also showed both thermal transitions with the *T*_g_ and *T*_m_ values corresponding to the syndiotactic P(M)MA and crystalline P3HT domains. With successful encapsulation of the acceptor C_60_ by graft copolymer *st*-P(M)MA-*g*-P3HT to construct a thermodynamically driven (via molecular recognition), unique supramolecular helical crystalline complex, these new architectures offer a novel strategy to potentially control the donor/acceptor domain morphology of critical importance to the solar conversion efficiency of OPV solar cells.

## Figures and Tables

**Table 1:** Results of homopolymerization of P3HT-(CH_2_)_2_-MA and its copolymerization with MMA *^a^*.

Run	Cat.	P3HT-MA Wt, *M*_n_ *^b^*	MMA (mg)	MMA/MA-end *^c^*	Yield (mg)	P3HT *^d^* (mol %)	*mm ^e^* (*rr*)	*T*_g_ *^f^* (°C)	*T*_m_ *^f^* (°C)	*M*_w_ *^g^* (kg/mol)	*Ð ^g^* (*M*_w_/*M*_n_)
1	Zr-**1^+^**	50, 5000	0	n/a	46	n/a	n/d	53.2	224	n/d	n/d
2	Zr-**1^+^**	30, 5000	230	383/1	210	4.3	92%	51.9	217	132	1.25
3	Zr-**1^+^**	30, 5000	23	38/1	45	13.2	94%	52.3	216	27.4	1.14
4	Zr-**2^+^**	50, 5000	0	n/a	48	n/a	n/d	132.8	219	n/d	n/d
5	Zr-**2^+^**	30, 5000	230	383/1	200	3.9	(95%)	111.9	218	43.2	1.32
6	Zr-**2^+^**	30, 3500	93	108/1	110	8.0	(94%)	n/d	n/d	33.1	1.15
7	Zr-**2^+^**	30, 3500	23	27/1	43	14.5	(93%)	n/d	n/d	19.5	1.16

*^a^* Conditions for homopolymerization (toluene, 25 °C): V(total) = 8.0 mL, time = 12 h, P3HT-MA/Zr = 1/1 (molar ratio); copolymerization (toluene, 25 °C): V(total) = 8.0 mL, time = 6 h, MMA/Zr = 20/1 (molar ratio). *^b^* P3HT-MA weight (Wt in mg) with two different molecular weights (*M*_n_). *^c^* Molar ratio of MMA to MA-end. *^d^* P3HT content measured by ^1^H-NMR spectra. *^e^* Isotacticity and syndiotacticity (numbers in parenthesis) measured by ^1^H-NMR spectra. *^f^* Melting-transition temperature (*T*_m_) and glass-transition temperature (*T*_g_) measured by DSC. *^g^* Weight-average molecular weight (kg/mol) and dispersity (*M*_w_/*M*_n_) determined by GPC.
